# Production and Analysis of Perdeuterated Lipids from *Pichia pastoris* Cells

**DOI:** 10.1371/journal.pone.0092999

**Published:** 2014-04-18

**Authors:** Alexis de Ghellinck, Hubert Schaller, Valérie Laux, Michael Haertlein, Michele Sferrazza, Eric Maréchal, Hanna Wacklin, Juliette Jouhet, Giovanna Fragneto

**Affiliations:** 1 Institut Laue-Langevin, Grenoble, France; 2 Service des polymères, Université Libre de Bruxelles, Brussels, Belgium; 3 Institut de Biologie Moléculaire des Plantes du CNRS, Strasbourg, France; 4 Laboratoire de physiologie cellulaire et végétale, CNRS/CEA/Univ. Grenoble Alpes/INRA, Grenoble, France; 5 European Spallation Source ESS AB, Lund, Sweden; 6 Chemistry Department, University of Copenhagen, Copenhagen, Denmark; Louisiana State University Health Sciences Center, United States of America

## Abstract

Probing molecules using perdeuteration (i.e deuteration in which all hydrogen atoms are replaced by deuterium) is extremely useful in a wide range of biophysical techniques. In the case of lipids, the synthesis of the biologically relevant unsaturated perdeuterated lipids is challenging and not usually pursued. In this work, perdeuterated phospholipids and sterols from the yeast *Pichia pastori*s grown in deuterated medium are extracted and analyzed as derivatives by gas chromatography and mass spectrometry respectively. When yeast cells are grown in a deuterated environment, the phospholipid homeostasis is maintained but the fatty acid unsaturation level is modified while the ergosterol synthesis is not affected by the deuterated culture medium. Our results confirm that the production of well defined natural unsaturated perdeuterated lipids is possible and gives also new insights about the process of desaturase enzymes.

## Introduction

Probing molecules using perdeuteration (i.e. deuteration in which hydrogen atoms are replaced by deuterium) is one of the most efficient methods for the investigation of the structure and dynamics of biological systems by means of NMR, infrared spectroscopy or neutron scattering. The striking differences in the scattering cross sections between hydrogen- and deuterium-containing compounds has made neutron scattering a very powerful tool in biophysical research in general and in membrane research in particular [1]. Isotope labelling allows enhancing the scattering signal of a molecule and/or to highlight the interesting parts of a system. With the possibility to resolve structural details from the fraction of a nanometer up to several hundred nanometers, neutron scattering is becoming more and more popular. The recent optimization of neutron instrument performance is such that often the bottleneck is the quality of the samples and their biological relevance.

Over the years isotopic substitution has been used to study viruses [1], [2], proteins [3], [4], [5], DNA/RNA structure [6], [7], [8]), and model membranes [9], [10], [11]. The development of dedicated protein deuteration facilities has greatly contributed to the current boosting of activities. With regards to structural biology, the various neutron [12] and X-ray scattering techniques complement crystallographic studies that require high quality crystals of macromolecules. For example, small angle neutron and X-ray scattering are widely used for the study of biomolecules and their assemblies in solution, including molecules difficult to crystallize. In parallel, neutron and X-ray reflectometry are widely used to characterize oriented structures on planar surfaces [13], [14]. These techniques all employ the contrast variation method, in which chemically equivalent structures with different isotopic content are analyzed simultaneously for enhanced resolution. Model bilayers prepared from synthetic lipids have been extensively investigated to understand structural features, phase behaviour, water permeability and changes induced by membrane proteins [15], [16], [17], but very few structures have been published for native cell membranes, or native-like lipid mixtures. While the model studies are useful to understand fundamental aspects of cell membrane behaviour, preparations of deuterated, naturally occurring lipid mixtures are necessary to elucidate real biological systems and to link models to cell membranes. Detailed knowledge of the membrane composition is a pre-requisite to the implementation of membranes that contain isotopically labelled components, as their analysis requires the use of chemically identical preparations. Separation of lipids into different classes further allows investigations of their particular effects on membrane properties and biological processes such as interaction with proteins, peptides and pharmaceuticals.

Commercially available perdeuterated lipids consist of either fully saturated or mono unsaturated phospholipids although poly-unsaturated chains also occur widely in nature as they are crucial for the lateral fluidity of natural membranes. While synthetic routes to obtain unsaturated chains are not trivial, it is possible to obtain enzymatically-produced perdeuterated unsaturated lipids by extracting them from living organisms as long as these organisms are capable to grow in a deuterated medium allowing the biosynthesis and efficient use of deuterated organic building blocks.

The methylotrophic yeast *Pichia pastoris* has been widely used as an expression system to produce biological compounds [18], [19]. Its ability to grow in fully deuterated medium has made it an exceptional system for the synthesis of perdeuterated macro molecules which are very difficult to synthesize chemically.

Production of perdeuterated heterogeneous materials by genetically modified yeast has been reported previously [20], [21], [22], but *Pichia pastoris* has not been extensively studied as a cell factory to produce deuterated lipids. The detailed pathways of fatty acid biosynthesis (including elongation and desaturation) and lipid regulation have however been investigated in this model organism [23], [24], [25]. A few studies report the role of deuterated environment on *Pichia* lipid biosynthesis [21], [22], [26], in comparison with available data on hydrogenated lipid metabolism and composition [27], [28], [29]. It turns out that growing *Pichia angusta* in deuterated environment with methanol as carbon source leads to an enhanced production of phosphatidylinositol (PI) [26].

With the aim to use naturally deuterated membranes in biophysical studies, we needed to check that lipids produced by *P. pastoris* cells in a deuterated environment were identical to those produced in a hydrogenated one. The analytical work presented here provides therefore the basis for the interpretation of data sets in future comparative studies of the structure and dynamics of hydrogenated and deuterated membranes using biophysical methods.

## Materials and Methods

### 1. Strains and culture conditions

*Pichia pastoris* GS115 HSA (Invitrogen) cells were grown at 30°C in 10 ml of BMGY medium 10 g/l yeast extract, 20 g/l peptone, 5.96 g/l KH_2_PO_4_, 1.07 g/l K_2_HPO_4_, 13,4 g/l yeast nitrogen base with ammonium sulphate without amino acids, 4 mg/l biotin, 10 g/l glycerol) in a 100 ml Erlenmeyer flask (Corning) using vent caps for continuous gas exchange with shaking at 250 rpm.

After 4 days 1 ml of this pre-culture was diluted into 100 ml of minimal medium ((38.1 g/l H_3_PO_4_, 0.93 g/l MgSO_4_, 4.13 g/l KOH, 20 g/l glycerol, 0.4 mg/l biotin, 40 mg/l histidine, 26 mg/l cupric sulphate pentahydrate, 0.35 mg/l sodium iodide, 13 mg/l manganese sulphate monohydrate, 0.87 mg/l sodium molybdate dehydrate, 0.09 mg/l boric acid, 2.17 mg/l cobalt chloride, 87 mg/l zinc chloride, 0.28 g/l ferrous sulphate heptahydrate, 87 mg/l biotin, 40 mg/l sulphuric acid; the pH was a adjusted to 6.0 using NH_4_OH) and incubated at 30°C for 2–3 days with shaking at 250 rpm.

The composition of PTM_1_ was the following: cupric sulphate pentahydrate 6 g/l, sodium iodide 0.08 g/l, manganese sulphate monohydrate 3.0 g/l, sodium molybdate dehydrate 0.2 g/l, boric acid 0.02 g/l, cobalt chloride 0.5 g/l, zinc chloride 20.0 g/l, ferrous sulphate heptahydrate 65.0 g/l, biotin 0.2 g/l, sulphuric acid, 5 ml/l.

For the adaptation of perdeuterated *Pichia pastoris*, 1 ml of the culture in minimum medium was diluted into 100 ml of perdeuterated basal salt medium (d-BSM) and incubated at 30°C for 5–6 days. The deuterated medium was prepared in the following way: 1 litre of hydrogenated basal salt medium (h-BSM) without glycerol was flash evaporated, the powder re-suspended in 250 ml of 99.85% D_2_O (Euriso-top) and flash evaporated again. This process was repeated twice to get rid of trace H_2_O. Finally the powder was re-suspended in 1 l D_2_O (purity >99.9%, from the Institut Laue-Langevin, France) containing 20 g d_8_-glycerol (Euriso-top). The deuterated culture was diluted again in d-minimal medium and grown for 2–3 days and used as inoculum for the final deuterated culture.

1 ml of each H- or D-pre-culture was taken to inoculate 150 ml of h-BSM and d-BSM and incubated at 18°C or 30°C. The initial optical cell density (OD) was approximately 0.3 for all the cultures.

To compare the composition of phospholipids at different temperatures and in isotopic growth media, *P. pastoris* cells were harvested by centrifugation at the early exponential phase, estimated by an OD_600_ of 20. To compare the content of sterol, cells were harvested at the late exponential phase.

### 2. Phospholipid analysis

Lipids were extracted from freshly harvested *P. pastoris* cells according to a modified Folch method. The modification consists in boiling freeze-dried cells in ethanol for five minutes in order to denature endogenic enzymes such as phospholipases capable of damaging glycerolipids. The rest of the extraction followed the Folch procedure [30].

Phospholipids were separated by two-dimensional thin layer chromatography (2D-TLC) using 20 cm×20 cm glass plates coated with silica (silica gel 60, Merck). The first chromatographic dimension was achieved by elution in chloroform∶methanol∶water (65∶25∶4, v/v), then the TLC plate was dried thoroughly under a stream of argon, and the second chromatographic dimension was performed in chloroform∶acetone∶methanol∶acetic acid∶water (50∶20∶10∶10∶5, v/v). Lipids were visualized under ultraviolet (UV) light after staining with 8-anilino-1-naphthalenesulfonic acid, 2% in methanol, and identified by comparison with standards.

Phospholipid spots were scraped off the TLC plates separately and known amounts of C21:0 fatty acid (Sigma) were added to each lipid spot as an internal standard. Fatty acids from the glycerolipids and the control C21:0 fatty acid were methylated by 1 h incubation at 100°C with 2.5% H_2_SO_4_ in pure methanol (3 mL total volume) in a sealed glass vial. Th reaction was stopped by the addition of 3 ml of water and 3 ml of hexane. Following the formation of a biphasic system, the upper phase containing the fatty acid methyl esters (FAMEs) was collected, dried under a stream of argon, re-suspended in pure hexane and then analyzed by gas chromatography with flame ionisation detector (GC-FID) (Perkin Elmer) on a BPX70 (SGE) column. The temperature program included 7 min 30 s at 130°C, then a ramp from 130°C to 180°C at 3°C/min and 10 min at 180°C. N_2_ was used as a carrier gas (3.5 mL/min). FAME retention times were compared with those of hydrogenated standard FAMEs (Sigma) and deuterated FAMEs. Deuterated FAMEs have a shorter retention time than the hydrogenated ones (figure S2). Standard deuterated C16:0 and C18:0 FAMEs were obtained by methanolysis of 1,2-dipalmitoyl(d62)-sn-glycero-3-phosphocholine and 1,2-distearoyl(d70)-sn-glycero-3-phosphocholine respectively (from Avanti) (figure S1).

### 3. Sterol analysis

The analytical process was performed as previously described [31], [32]. Freeze-dried *Pichia* cells (50 to 100 mg) were saponified for 2 h at 80°C in a methanolic potassium hydroxide solution (6%, w/v). The unsaponifiable part was extracted three times with *n*-hexane. The dried residue was analyzed by GC-MS using a 6890 gas chromatograph (Agilent) equipped with an HP5-MS column (30 m long, 0.32 mm i.d., 0.25 mm film thickness) coupled with a 5973 mass selective detector (Agilent). The temperature program included a ramp from 60°C to 220°C (30°C/min) then a ramp from 220°C to 300°C at 2°C/min. Helium was used as a carrier gas (2 mL/min). Mass spectrometry of sterols was performed as described previously [33], [34]. Sharp peaks of ergosta-5,7,22-trienol (ergosterol) were resolved as acetate derivatives. For this purpose, dry hexane extracts were incubated with a mixture (100 µL) (2∶1, v/v) of acetic anhydride∶pyridine for 30 min at 70°C. Reagents were then dried off. The resulting ergosteryl acetate and deuterioergosteryle acetate contained therefore 3 hydrogen atoms of non-biosynthetic origin.

## Results

### 1. Cell growth

**Figure 1:**
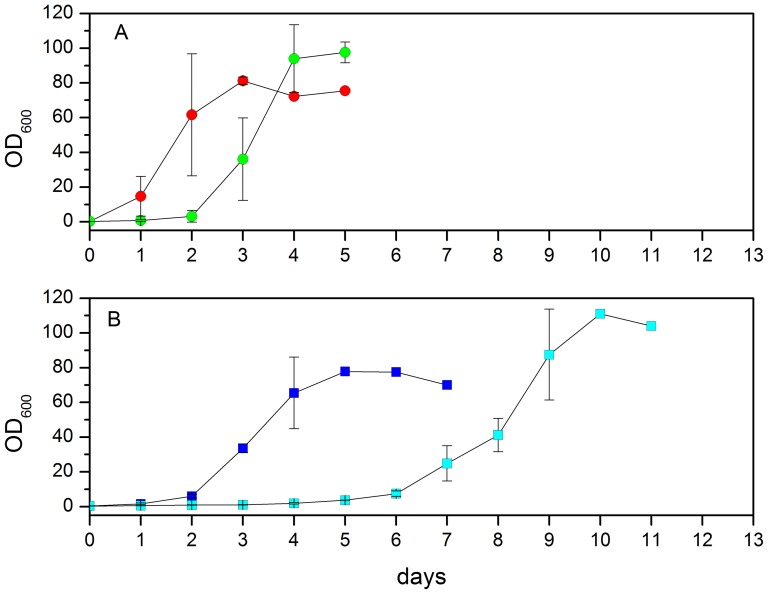
Growth curves of *Pichia Pastoris* cells. (A) Growth of *Pichia pastoris* cells in a hydrogenated medium at 30°C (red circle) and 18°C (green circle). (B) Growth of *P. pastoris* cells in a deuterated medium at 30°C (blue square) and 18°C (cyan square). Errors bars represent the standard deviation from three independent growth curves.

Figure 1 shows the growth curves of yeast cells at 18°C and 30°C in (A) hydrogenated and (B) deuterated media. In hydrogenated (H) medium, the cells immediately started to grow from the first day at 30°C, whereas there was a lag of two days when H cells were grown at 18°C. The growth rate in the logarithmic phase was the same for both temperatures. In deuterated (D) medium, the lag phase was longer: it took two days for the D yeast cells to grow at 30°C and 5 days at 18°C. Like H yeast cells, the lag phase of D yeast cells is longer when they are grown at 18°C. In both hydrogenated and deuterated growth media, the growth rate is not limited by temperature.

### 2. Influence of deuterated environment on phospholipid class and total fatty acid distribution

**Figure 2:**
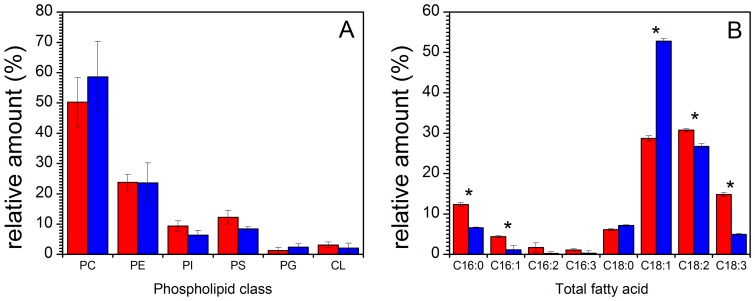
Phospholipid and fatty acid composition of *Pichia Pastoris* cells. (A) Phospholipid composition and (B) total fatty acid distribution of *Pichia pastoris* cells grown in a hydrogenated medium (red) and in a deuterated environment (blue) at 30°C. PC, phosphatidylcholine; PE, phosphatidylethanolamine; PI, phosphatidylinositol; PS, phosphatidylserine; PG, phosphatidylglycerol; CL, cardiolipin or diphosphatidyglycerol. Data represent mean values ± s.d (n = 3). In histograms, *P<0.05 from Student's *t*-test, assuming equal variance.

Phospholipid composition of *P. pastoris* cells grown in hydrogenated medium with glycerol as a carbon source is consistent with previous reports [35]. No significant changes in phospholipid class composition were observed when yeast cells were grown in a deuterated environment, as shown in figure 2A which shows the relative amounts of the main phospholipids, mainly PC, phosphatidylcholine; PE, phosphatidylethanolamine; PI, phosphatidylinositol; PS, phosphatidylserine; PG, phosphatidylglycerol; CL, cardiolipin or diphosphatidyglycerol. We conclude that the various lipid class metabolic pathways were not affected by the isotopic conditions of the growth medium.

It is however noticeable that the deuterated growth medium has an influence on the acyl carbon chains, in terms of lengths and unsaturation levels (figure 2 B). The most obvious feature is the remarkable increase of C18:1 chains. The ratio of C16/C18 fatty acids is also affected when the cells are grown in a deuterated environment. This ratio is 0.24±0.01 in H-lipids and 0.11±0.02 in D lipids (see table 1). Therefore the deuterated environment is likely to have an influence on the activity level of elongase enzymes. Although we cannot assess whether the effect is due to a decrease of the expression levels of the elongase genes, the appropriate folding of the enzymes to catalyze elongation efficiently, or an alteration of their activity for deuterated substrates, it is reasonable to suggest that the enzyme specificity for its substrates might be sensitive to the presence of deuterium in place of hydrogen. The ratio of unsaturated/saturated fatty acids (UFA/SFA) is 4.42±0.20 in a hydrogenated medium and 6.28±0.28 in a deuterated medium (table 1 and figure S2). This change mainly reflects the enhanced production of C18:1 in deuterated cells (see figure 2). It is worth noting that the production of deuterated C18:3 is highly inhibited in deuterated medium while that of C18:2 is somewhat inhibited.

**Table 1:** Ratio (upper part) of C16/C18 fatty acids and (lower part) of unsaturated/saturated fatty acid (UFA/SFA) when *Pichia pastoris* cells were grown in hydrogenated (H) and deuterated (D) media at 18 and 30°C.

C16/C18	Total^a^	PC^a^	PE^a^	PI^a^	PS^a^	CL^a^	PG^a^
H 30°C	0.24±0.01	0.23±0.01	0.27±0.01	0.53±0.02	0.67±0.02	0.09±0.02	0.60±0.02
H 18°C	0.14±0.03	0.12±0.01	0.20±0.03	0.43±0.08	0.52±0.02	0.08±0.01	0.22±0.32
D 30°C	0.11±0.02	0.05±0.01	0.18±0.02	0.38±0.03	0.53±0.03	0.18±0.14	0.92±0.38
D 18°C	0.18±0.02	0.09±0.01	0.27±0.02	0.48±0.03	0.65±0.02	0.21±0.12	0.61±0.29

UFA/SFA	Total	PC	PE	PI	PS	CL	PG
H 30°C	4.42±0.20	6.34±0.20	3.60±0.10	1.52±0.10	1.32±0.03	8.81±3.64	1.22±0.20
H 18°C	5.70±0.22	25.89±4.86	5.14±0.87	1.92±0.45	1.52±0.05	11.03±0.65	1.65
D 30°C	6.28±0.13	17.16±1.23	5.56±0.17	2.08±0.32	1.69±0.08	11.86±9.11	1.64±0.28
D 18°C	6.19±0.02	15.39±1.41	4.14±0.21	2.17±0.18	1.88±0.03	10.36±1.88	1.86±0.03

a : Total, total fatty acids; PC, phosphatidylcholine; PE, phosphatidylethanolamine; PI, phosphatidylinositol; PS, phosphatidylserine; PG, phosphatidylglycerol; CL, cardiolipine.

### 3. Influence of temperature on total fatty acid production

It is known that yeasts can regulate the fatty acid distribution to keep the membrane fluidity constant by increasing the production of polyunsaturated fatty acids (PUFA) if the temperature is lowered during cultivation [36]. We investigated this physiological response by decreasing the temperature of a culture grown at 30°C to 18°C to check whether it was possible to modulate the production of deuterated PUFA using the physiological machinery controlling fatty acid quality in yeast.

In hydrogenated conditions, the proportion of PUFAs increased concomitantly with the C18/C16 ratio. This result is consistent with previous studies [36].

**Figure 3:**
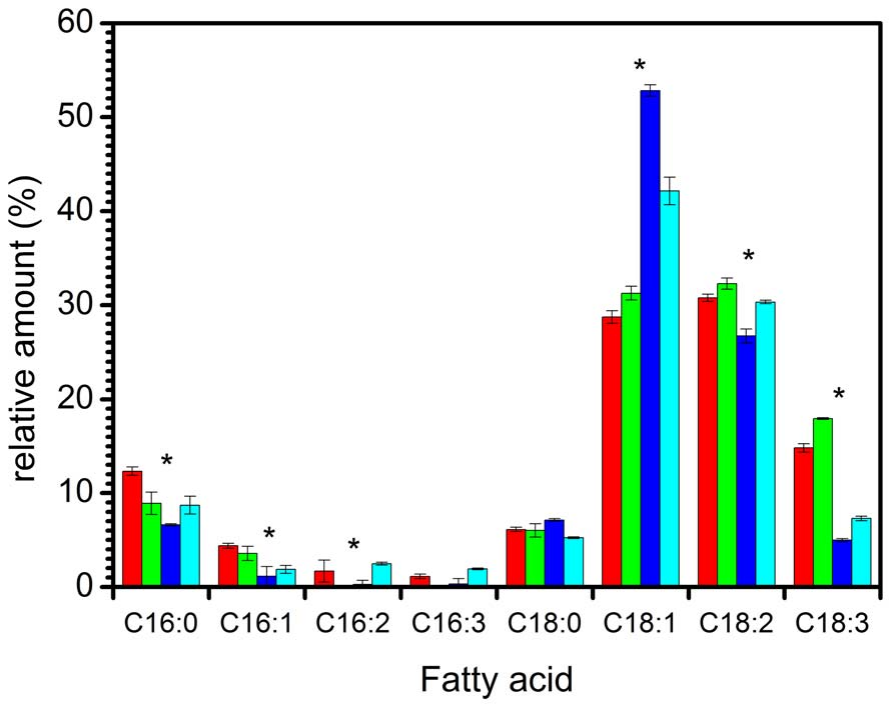
Total fatty acid distribution in *Pichia pastoris* cells grown in a hydrogenated environment at 30°C (red) and 18°C (green) and in a deuterated environment at 30°C (blue) and 18°C (cyan). Data represent mean values ± s.d (n = 3). In histograms, *P<0.05 from Student's *t*-test, assuming equal variance. For C16:0, there is a significant difference between H 30°C and H 18°C, between H 30°C and D30°C, H 30°C and D18°C, between H 18°C and D 30°C but not between H18°C and D18°C. For C16:1, there is a significant difference for all 4 different temperatures and isotopic contents. For C16:2, there is a significant difference between D30°C and D18°C. For C18:1, there is a significant difference for all 4 different temperatures and isotopic contents. For C18:2, there is a significant difference between D30°C and H30°C, between D30°C and D18°C, between D30°C and H18°C. For C18:3, there is a significant difference for all 4 different temperatures and isotopic contents.

Deuterated yeast cells also increased their production of PUFA when grown at 18°C, although less than the corresponding hydrogenated control (figure 3). The proportion of deuterated C18:1 decreased from 53% at 30°C to 43% at 18°C, while the proportion of deuterated fatty acid C18:2 increased from 27% to 30% and fatty acid C18:3 from 5% to 7%. The production of C18:1, C18:2 and C18:3 all increased in hydrogenated yeast when lowering growth temperature.

The ratio C16/C18 of deuterated yeast extracts at 30°C was 0.11±0.02 while it was 0.18±0.02 at 18°C. Surprisingly, a change in the opposite direction occurred in the hydrogenated batch, from 0.24±0.01 at 30°C to 0.14±0.02 at 18°C (table 1).

### 4. Fatty acid analysis of the different classes of phospholipids

**Figure 4:**
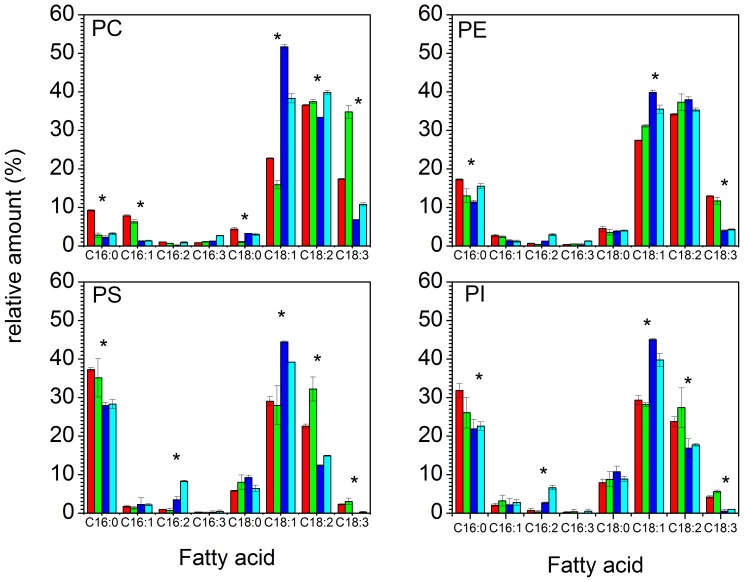
Fatty acid distribution of the main phospholipids produced by *Pichia pastoris* cells grown in a hydrogenated medium at 30°C (red) and at 18°C (green) and in a deuterated environment at 30°C (blue) and 18°C (cyan). In all individual phospholipids, the deuterated environment triggers the enrichment in C18:1 fatty acids PC, phosphatidylcholine; PE, phosphatidylethanolamine; PI, phosphatidylinositol; PS, phosphatidylserine. Errors bars represent the standard deviation from three different phospholipids extractions and separations. Data represent mean values ± s.d (n = 3). In histograms, *P<0.05 from Student's *t*-test, assuming equal variance. PC: for C16:0, the difference is significant between H30°C and H18°C, between H30°C and D30°C and between H30°C and D18°C. For C16:1, there is a significant difference between H30°C and D30°C and between H18°C and D18°C. For C18:0, there is a significant difference between H18°C and D18°C, between H18°C and H30°C and between H18°C and D30°C. For C18:1, the difference is significant between all the different temperatures and isotopic contents. For C18:2, there is a significant difference between D30°C and D18°C, between D30°C and H30°C and between D30°C and H18°C. For C18:3, the difference is significant between all the different temperatures and isotopic contents. PE: for C16:0, the difference is significant between H30°C and D30°C, between H30°C and H18°C and between H30°C and D18°C. For C18:1 and C18:3, the difference is significant between all the different temperatures and isotopic contents. PS and PI: for C16:0, there is a significant difference between H30° and D30°C and between H30° and D18°C. For C16:2 and C18:1, there is a significant difference between H30°C and D30°C, between H18°C and D18°C and between D18°C and D30°C. For C18:2 and C18:3, there is a significant difference between H30°C and D30°C and between H18°C and D18°C.

Some of the general patterns found in the global composition of fatty acyl chains of all the glycerolipids (here called total fatty acids) were also present in individual phospholipid class fatty acid distributions (figure 4). 1) At 30°C in deuterated conditions, C18:1 is always the main fatty acid in all lipid classes. 2) Deuterated C18:3 synthesis is always inhibited in comparison to C18:3 generated in a hydrogenated medium. 3) Lowering the temperature enhances the production of deuterated polyunsaturated fatty acids (PUFA), but not enough to reach the level of PUFAs in hydrogenated yeast. 4) The C16/C18 ratio of deuterated fatty acids increases while the C16/C18 ratio of hydrogenated fatty acids decreases by lowering the growth culture temperature (see table 1).

By examining more carefully the fatty acid distributions in each class of phospholipids, we can highlight more subtle differences that might reflect some specific effects on enzymatic activities when using deuterated substrates.

In phosphatidylcholines, the ratio of hydrogenated C16/C18 decreases when the growth medium is cooled down, whereas the opposite trend is observed in deuterated cells. The ratio of unsaturated to saturated fatty acids (UFA/SFA) of hydrogenated PCs is much lower than that of deuterated PCs at 30°C (table 1). When the growth media is cooled down from 30°C to 18°C, the UFA/SFA ratio in hydrogenated media dramatically increases from 6.34±0.20 at 30°C to 25.89±4.86 at 18°C. This very high ratio comes from the fact that, in PC, almost no hydrogenated C16:0 or C18:0 are present at 18°C. In deuterated PCs, the growth temperature does not affect the UFA/SFA ratio. It is worth noticing that, in hydrogenated conditions, the production of C18:3 increased from 18 to 35% when lowering the growth temperature to 18°C.

Phosphatidylethanolamines exhibits less variation as function of the isotopic or thermal conditions compared to the other phospholipids. When lowering growth temperature, the C16/C18 ratio decreases in hydrogenated cells and increases in deuterated cells and the UFA/SFA ratio increases in hydrogenated cells and decreases in deuterated cells. However, these differences are minor and the C16/C18 and SFA/UFA ratios are roughly identical at both growth temperatures and isotopic conditions. The most remarkable feature is the inhibition of C18:3 synthesis in the deuterated culture, which is a common feature observed in all other phospholipids.

The C16/C18 and UFA/SFA ratios in phosphatidylinositol (PI) and phosphatidylserine (PS) follow the same general trends. It is worth noting that PS and PI have a very similar fatty acid distribution. In deuterated growth conditions, the amounts of C16:0 and C18:2 are reduced and C18:3 almost completely vanished. However, the production of C16:2 is triggered in deuterated media and is amplified by lowering growth temperature.

### 5. Ergosterol biosynthesis in a deuterated environment

**Figure 5:**
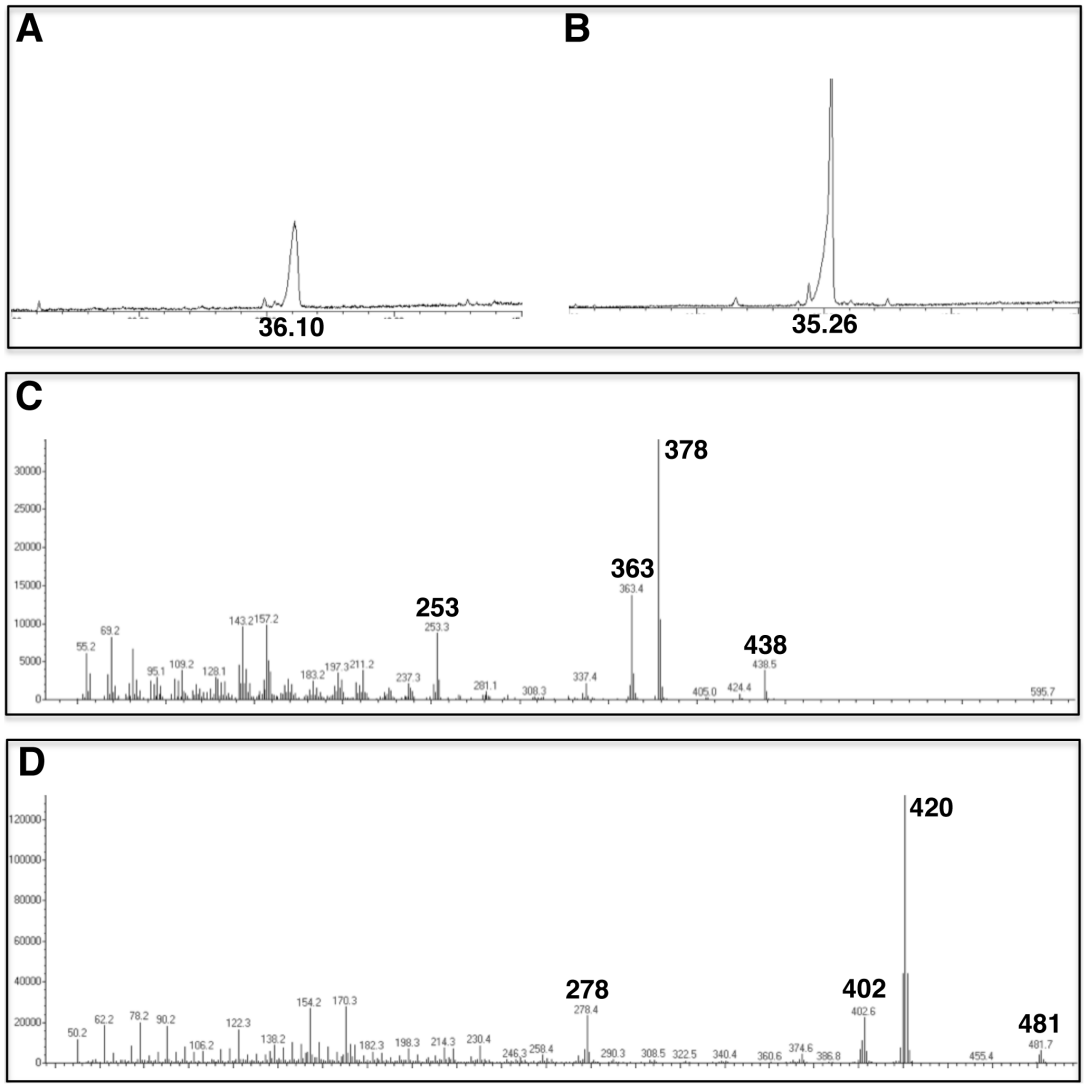
GC-MS analysis of ergosterol isotopomers from *Pichia pastoris* unsaponifiable extracts. A, TIC of an acetylated extract from cells grown in hydrogenated medium. The peak at RT = 36.10 min is ergosterol. B, TIC of an acetylated extract from cells grown in deuterated medium. The peak at RT = 35.26 min is deuterioergosterol. C, mass spectrum of ergosteryl acetate. Prominent ions and interpretation of the fragmentation pattern: M^+^(438), M^+^-acetate-H (378), M^+^-acetate-H-CH_3_ (363), M+-acetate-H-side chain (253). D, mass spectrum of deuterioergosteryl acetate. Prominent ions and interpretation of the fragmentation pattern: M^+^(481), M^+^-acetate-D (420), M^+^-acetate-D-CD_3_ (402), M^+^-acetate-D-side chain (278).

*Pichia pastoris* cells grown in hydrogenated or in deuterated media contained ergosterol and d-ergosterol, respectively, as their major sterol constituents (Figure 5). Trace amounts of ergostatetra-5,7,22,24(28)-enol and lanosterol were barely detectable. GC traces showed indeed a single peak of ergosteryl acetate (Figure 5A), and of d-ergosteryl acetate (Figure 5B). The deuterated compound had a shorter retention time (of about one minute) than the hydrogenated one. The molecular fragmentation of light and heavy isotopomers of ergosteryl acetate performed by electron impact at 70 eV clearly demonstrated the biogenesis of fully deuterated ergosterol by *Pichia pastoris* grown in the presence of d_8_-glycerol as a sole carbon source. Indeed, molecular ions at m/z 438 in the case of ergosteryl acetate (Figure 5C), and at m/z 481 in the case of deuterioergosteryl acetate (Figure 5D), confirmed the formula C_30_H_46_O_2_ and C_30_D_43_H_3_O_2_ for the analyzed isotopomers. In addition, GC-MS analysis of the underivatized extract revealed molecular ions at m/z 396 or at m/z 440 (spectra not shown), in full agreement with ergosterol (C_28_H_44_O) or deuterioergosterol (C_28_D_44_O), respectively.

## Discussion

### 1. Desaturation of fatty acid in yeast

In yeasts, lipids are mainly composed of C16 and C18 fatty acids which are usually produced through *de novo* synthesis, even though yeasts can assimilate exogenous fatty acids when supplied in the growth medium [37]. Acyl-CoA is the first substrate for unsaturation at the level of carbon alpha-9 via the action of a Δ9-desaturase. Δ12 and Δ15 desaturase enzymes catalyse the addition of a second and third double bond on the fatty acid chains when esterified to a glycerolipid. Evidence of the presence of these ubiquitous desaturases in yeasts has been reported [38].

In *Saccharomyces cerevisiae*, lipids are mainly composed of C18:1 and C16:0 [23]. However this is not the case in all yeast species. In *P. pastoris*, it has been shown that the relative amount of polyunsaturated fatty acids is much higher [28], [29]. This pattern was also found in the present work. In comparison, *Pichia pastoris* cells grown in a deuterated environment produce mainly C18:1 at the expense of C18 polyunsaturated fatty acids and C16 fatty acids. This finding is consistent with the fact that yeasts can regulate the production of PUFA to control the membrane fluidity in stress conditions [36].

The UFA/SFA ratio is in a steady state and is determined by the competition of the enzymes Ole1p and Sctp1p on the C16:0 acyl-CoA substrate. Ole1p catalyzes the introduction of the first double bond in the aliphatic chain. Besides, Sctp1p can shield the acyl-CoA substrate to prevent the desaturation triggered by Ole1p. The isotopic substitution may have an effect on the mutual balance of this set of enzymes [39].

Adding a double bond in a fatty-acyl substrate is a very demanding process that involves two electrons and the cleavage of two C-H bond (98 kCal/mol). This reaction consumes molecular oxygen. In nature, there are two clearly identified kinds of Δ9 desaturase enzymes which introduce the first double bond, in cis or Z conformation, at the alpha-9 carbon. The first one is a plant-specific soluble desaturase and the second one is an integral membrane desaturase found in animals and fungi [23].

The Kinetic Isotopic Effect (KIE) consists in replacing a CH_2_ by a CD_2_ unit in different positions in the fatty acid chain and measuring the kinetics of the desaturation process. It has been shown [40], [41] that when deuterium substitutes hydrogen bound to carbon in position 9, the constant rate of the desaturation reaction is reduced by a factor of 7, whereas deuterium substitution on carbon 10 does not affect the desaturation rate. This shows that the desaturation occurs in two steps. It also means that the presence of deuterium in a particular location can highly slow down the desaturation reaction.

This KIE is consistent with our *in vivo* experiments reported here, and provides clues to understanding why the amounts of C18:2 and C18:3 in deuterated fatty acid were low compared to that of C18:1. If deuterium substitution slows down the introduction of one double bond, one can expect that it has the same effect for the addition of supplementary double bonds. Therefore the action of the Δ12 and Δ15 desaturase enzymes might be slowed down because of the fully deuterated chains, consequently C18:1 is accumulating in the fatty acid pool. However, decreasing the culture medium temperature also has the consequence of slowing down the growth rate of yeast cells. In this condition, the growth rate decrease compensates partially for the reduced activity rate of Δ12 and Δ15 enzymes, but this effect is not strong enough to produce as much PUFA as in the hydrogenated species.

Therefore, we can conclude that the increased production of C18:1 probably comes from two types of contribution: the regulation of yeast to maintain the membrane fluidity and the kinetic isotopic effect. The effect of temperature can be comprehended by comparing the fatty acid composition of the h-yeast at 30°C and that of h-yeast at 18°C. The amount of C18:0 is not significantly changed when lowering the growth culture temperature. However, the amount of C18:1 and C18:2 increases by 1.5% and the amount of C18:3 increases by 3%. The effect of temperature in d-yeast results in a decrease of C18:0 of 2% and of C18:1 of about 10% and an increase of C18:2 and C18:3 of about 2.5%. Therefore, in both h and d-yeast, lowering the growth culture temperature results in the enhanced production of PUFA. To understand the KIE, it is more relevant to compare the fatty acid composition of h-yeast with that of d-yeast, both at 30°C. The dramatic increase of C18:1 of 24% is the most obvious effect, although a decrease of C18:2 of 4% and C18:3 of 10% can be observed. We can also analyse the KIE at 18°C. In this case, the increase of C18:1 between h-yeast and d-yeast is 11%, which is less pronounced than the KIE occurring at 30°C. Interestingly, the amount of C18:2 and C18:3 increased both by 3% in deuterated yeast, confirming that KIE decreased when lowering temperature.

### 2. Glycerophospholipid metabolism and regulation

The major phospholipids in yeasts are PC, PE, PI, PS, PG and CL. Some minor components include phosphatidic acid (PA), phosphatidyl-monoethyl-ethanolamine and phosphatidyl-diethyl-ethanolamine [25]. In *de novo* biosynthetic pathways, PA is the precursor for CDP-DAG (cytidine diphosphate-diacylglycerol). CDP-DAG is the intermediate for the production of PI, CL and PS. In this route, PE is made from PS *via* the action of two decarboxylase enzymes. PE then undergoes 3 successive methylations to generate PC. Alternatively, PC and PE can also be synthesized through the Kennedy pathway [42], [43]. Different cytosolic phosphorylase enzymes convert choline and ethanolamine into choline-P and ethanolamine-P. These intermediates are activated with cytidine triphosphate (CTP) resulting into choline-CDP and phosphoethanolamine-CDP. Finally these two activated substrates react with DAG generated from PA to form respectively phosphatidylcholine and phosphatidylethanolamine [25]. Both *de novo* and Kennedy pathway are functional in wild type yeast cells even in the absence of choline or ethanolamine in the growth medium [44]. PS, PI, PG and CL are synthesized through *de novo* pathway whereas PC and PE synthesis results from a combination of *de novo* and Kennedy pathways [45].

The similar fatty acid composition of PI and PS at different growth temperatures and isotopic media is explained by the fact that they are produced through the same pathway. However, the fatty acids patterns in PC and PE are different. Furthermore, the PC fatty acid pattern is more affected by the deuteration than the other phospholipid fatty acid patterns. This suggests that there might be a regulation of phospholipids happening downstream of fatty acid neo-synthesis at still uncharacterized levels of glycerolipid metabolism and probably at the PC level because PC is as an intermediate for all phospholipid synthesis via recycling of its diacylglycerol backbone in the other phospholipid classes [45].

Our results are different from those by Massou *et al.* [26], who observed an increase of PI which becomes the main phospholipid in deuterated conditions. However, they used different growth media and methanol as carbon source, which could have an impact on phospholipids biosynthesis. It has been shown that, in particular cases, such as nutrient depletion, the production of PI can be enhanced at the expense of PC [46]. Therefore, the difference in phospholipid composition in their study and ours could come from the change of carbon source or of media ion composition.

### 3. Biophysical effects of the fatty acid composition and challenges of using naturally occurring lipids in structural/functional studies

The lipid fatty acid composition is important for determining the physical properties of the membrane, such as the fluidity and hydrophobic core thickness which are crucial for regulating the interaction of, for example, cell penetrating peptides, pharmaceutical or other membrane active agents. It is therefore of critical significance to ensure that isotopically labelled membranes present the required composition when used in functional studies. In addition to this, deuteration is used in many scattering-based techniques in order to allow simultaneous analysis of several isotopic contrasts, when it is essential that the structures are chemically equivalent. In this case we have observed that the deuterated lipids have an increased C18:1 fatty acid content at the expense of a lower overall degree of polyunsaturation, but that this effect can be to some extent mitigated by lowering the growth temperature of the deuterated culture. Thus, isotopically labelled membranes show great promise as contrast agents for scattering based studies, provided that the lipid composition is analysed and that the isotope effects are taken into account in the growth conditions.

## Conclusions

The aim of this work was to optimize ways for producing biologically relevant models of cell membranes for biophysical studies by using deuterated lipids that are difficult to prepare via synthetic chemistry routes. The ability of the yeast species *Pichia pastoris* to grow in fully deuterated environment has been widely exploited in expression systems for producing fully deuterated proteins [22]. Therefore we used this system to produce deuterated unsaturated glycerolipids which are not commercially available. Our study highlights that the deuterated growth medium does not affect the general *P. pastoris* phospholipid class homeostasis. However, the fatty acid composition in each class of lipids is different to that observed in the cells grown in a hydrogenated medium. An accumulation of C18:1 fatty acid is strikingly triggered in deuterated lipids. Lowering the temperature reduces this effect but the amount of PUFA does not reach the level obtained in hydrogenated yeast. Based on our *in vivo* analyses and on previously published *in vitro* characterization of desaturases, this work suggest that *Pichia pastoris* is a model of choice as a cell factory for deuterated glycerolipids and that the production of deuterated PUFA might be improved by lowering the growth temperature and by the expression of heterologous genetic sequences coding desaturase enzymes, as reported in hydrogenated *P. pastoris* [47], [48], being less sensitive to a deuterated substrate.

## Supporting Information

Figure S1 **GC-FID spectra of fatty acid methyl esters (FAMEs).** A- hydrogenated standard containing C16:0, C16:1, C18:0 and C18:1 FAMEs (Sigma), B- deuterated C16:0 FAME and hydrogenated C21:0 FAME, C- deuterated C18:0 FAME and hydrogenated C21:0 FAME, D- FAMEs from hydrogenated C21:0 and total lipid extract of *P. pastoris* cells grown at 30°C in hydrogenated media, E- FAMEs from hydrogenated C21:0 and total lipid extract of *P. pastoris* cells grown at 30°C in deuterated media. Deuterated FAMEs have a shorter retention time than hydrogenated ones.
(PPTX)

Figure S2 **C18 saturated, mono- and poly-unsaturated fatty acid composition.** Total fatty acid are extracted from yeast grown in a hydrogenated environment at 30°C (red) and 18°C (green) and in a deuterated environment at 30°C (blue) and 18°C (cyan). Data represents mean values ± s.d. (n = 3).
(TIF)
