# Supplementary figures and images for: Production and Analysis of Perdeuterated Lipids from Pichia pastoris Cells
Source: PLoS One. 2014 Apr 18;9(4):e92999. doi: 10.1371/journal.pone.0092999 (PMC3991571; doi:10.1371/journal.pone.0092999)

## Slide 1
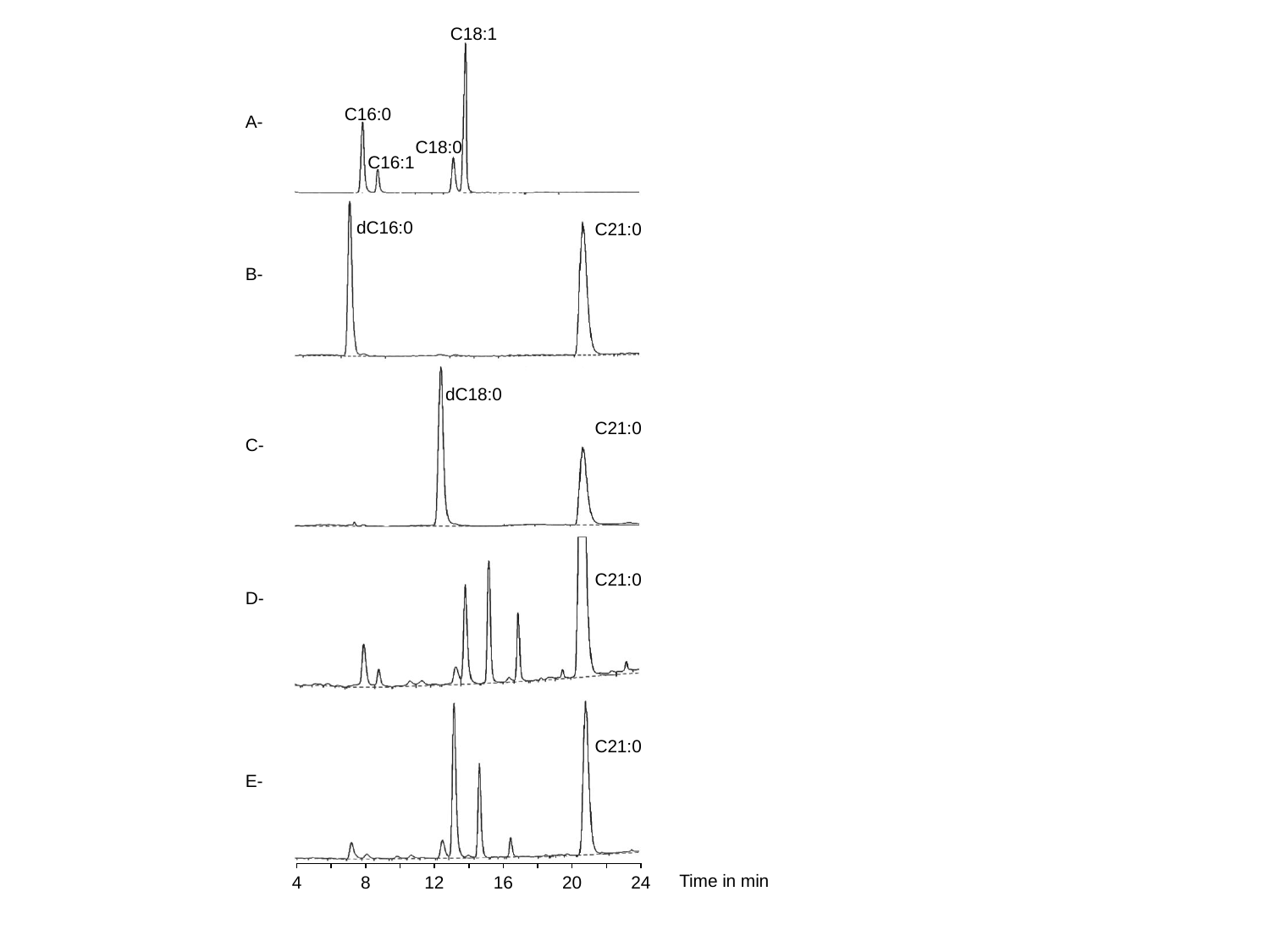

C18:1
C16:0
A-
C18:0
C16:1
dC16:0
C21:0
B-
dC18:0
C21:0
C-
C21:0
D-
C21:0
E-
4
8
12
16
20
24
Time in min

Supplement: Figure S1 — GC-FID spectra of fatty acid methyl esters (FAMEs). A- hydrogenated standard containing C16:0, C16:1, C18:0 and C18:1 FAMEs (Sigma), B- deuterated C16:0 FAME and hydrogenated C21:0 FAME, C- deuterated C18:0 FAME and hydrogenated C21:0 FAME, D- FAMEs from hydrogenated C21:0 and total lipid extract of P. pastoris cells grown at 30°C in hydrogenated media, E- FAMEs from hydrogenated C21:0 and total lipid extract of P. pastoris cells grown at 30°C in deuterated media. Deuterated FAMEs have a shorter retention time than hydrogenated ones. (PPTX) [file pone.0092999.s001.pptx]
